# A Lesson to Parents

**Published:** 1863-10

**Authors:** 


### A LESSON TO PARENTS.

I HAD been married fifteen years. Three beautiful daughters enlivened the domestic hearth, the youngest of whom was in her eighth year. A more happy and contented household was no where to be found. My wife was amiable, intelligent, and contented. We were not wealthy; but Providence had preserved us from want; and we had learned that "contentment without wealth, is better than wealth without contentment."

It was my custom, when returning home at night, to drop into one of the many shops that are constantly open in the business streets of the metropolis, and purchase some trifling dainties, such as fruit or confectionery, to present to mother and the children. I need not say how delighted the little ones were at this slight expression of paternal consideration. On one occasion I had purchased some remarkably fine apples. After the repast, half a dozen were left untouched, and my thrifty companion forthwith removed them to the place of deposit, where it was her custom to preserve the remains of our nick-nacks. A day or two after, when I had seated myself at the table to dine, she said to me smilingly:

"So, father has found the way to my safety-box, has he?"

I was at a loss to understand the meaning, and desired her to explain.

"Have you not been in my drawer?"

"What drawer?"

"The upper drawer in my chamber bureau. Did you not take therefrom the largest of the pippins I had put away for the girls?"

"No—I did not!"

"You did not?"

"Not I! I have not seen an apple since the evening I purchased them."

A slight cloud passed over the countenance of my wife. She was troubled. The loss of the apple was in itself nothing; but we had carefully instructed our children not to appropriate to their use, any article whatever of family consumption, without permission; and as permission, when the demand was at all reasonable, had never been denied them, she was loth to suspect any one of them of the offense. We had a servant-girl in the family, but as she was supposed to know nothing of the apples, my wife hesitated to charge it upon her. She at length broke the silence by saying:

"We must examine the affair. I can hardly think one of the children would so act. If we find them guilty, we must reprove them. Will you please look into it?"

The girls were separately called into my presence; the eldest first.

"Eliza, did you take from your mother's drawer, an apple?"

"No, sir."

"Maria, did you take from your mother's drawer, an apple?"

"No, sir."

"Mary, did you take from your mother's drawer, an apple?"

"No, sir."

"It must have been taken by the servant; call her to me," said I, addressing my wife.

"Nell, how came you to take from the drawer of your mistress, without permission, the largest of the apples she had placed there?"

"Wot apples?"

"Did you take no apple from the drawer of your mistress?"

"No sa."

Now, it was evident that falsehood existed somewhere. Could it be that one of my children had told me a lie? The thought harassed me. I was not able to attend to business. I went to the store—but soon returned again. Meanwhile, the servant-girl had communicated to her mistress that she had seen our youngest go into the garret with a large apple, the morning before. On examination, the core, and several pieces of the rind were found upon the floor. I again called Mary to me, and said to her affectionately:

"Mary, my daughter, did you not go into the garret yesterday?"

"Yes, sir."

"Did you go there with an apple?"

"No, sir."

"Did you notice any thing on the floor?"

"No, sir."

I was unwilling to believe my sweet child capable of telling me a falsehood; but appearances were against her. The fault lay between her and the servant, and while I was desirous to acquit my child, I did not wish to accuse unjustly the negro. I therefore took Mary into a room alone, I spoke to her of the enormity of lying—of the necessity of telling the truth—of the severe punishment I should be compelled to inflict upon her, if she did not confess the whole to me, and with tears in my eyes urged her to say that she had done it, if indeed she had. Gradually, I became convinced of her guilt; and now I felt determined she *should* confess it. My threatenings were not without effect. After weeping and protesting her innocence, and weeping and again protesting, my threatenings seemed to alarm her, and falling upon her knees, she said: "Father, I *did* take the apple."

Never shall I forget that moment. My child confessed that she was a liar, in my presence!

Suppressing my emotion, I retired; and Mary, rising from her position, ran to her mother, and in a paroxysm of grief cried out:

"Mother, I did *not* take the apple. But father has made me confess that I did."

Here was a new aspect of affairs. Lie multiplied upon lie. Could it be possible! My dear Mary, who had never been known to deceive us—so affectionate—so gentle—so truthful in all the past—could it be possible that she was a confirmed liar! Necessity was stronger than the tenderness of the father. I chastised her for the first time in my life—severely, severely chastised her! It almost broke her heart—and I may add, it almost broke mine also.

Yet Mary was innocent! After-events proved that the negro was the thief. She had conjured up the story of the garret, knowing that Mary would not deny having been there, and to make the circumstances strong against her, had strewn apple-rinds on the floor. I never think of the event without tears. But it has taught me a useful lesson, and that is never to threaten a child into a lie, when it may be he is telling the truth. The only lie I ever knew Mary to tell me, I myself forced upon her by threatenings. It has also fixed in my mind the determination to employ no servant in my family, when I can possibly do without.

The foregoing is a continuation of the article on Parental Corrections in the September number. The author is unknown, but if such impressive lessons have their due effect on the minds of parents, it will save many a pang in after years. "I read your September article on Parental Corrections," said a sunny-faced, energetic business man the other day. "I had just such a case in my own family. The mother was extremely impatient at the child; but I took him to his room, soothed his spirits as much as possible, and slept with him in his own little bed all night." A neighbor of ours, one of the very best of men in all the relations of domestic, social, and business life, corrected a little son of his with great harshness and severity. The next day the boy was taken ill, was sick for a long time, and barely escaped the grave. In the apprehension of the death of the child, and in the contemplation of his daily sufferings, he endured such inexpressible mental torture, he declared he would never punish a child of his again. This was going to the opposite extreme. It is seldom wise, in any domestic management, to lay down a Medo-Persian law; to pass any irrevocable edict; to make any unchangeable regulation. Such things are unbecoming in themselves, and indicate a weak mind. We are the creatures of circumstances. It is best to cultivate force of character, and leave ourselves free to act according to the exigencies of the moment. In reference to any action, whether good or bad, there is so much to modify it which can not be foreseen, that the highest wisdom is to leave one's self free to act when the time for action comes, steadily aiming to avoid haste and harshness; seeking a discreet medium between leniency and sternness; between license and liberty. But in reference to all our dealings with our children, knowing how fallible we are; knowing the numerous sources of misinformation and mistakes around us, it is well, if error must be committed, that it should be on the side of patience, forbearance, and a loving heart. Our children are unresisting, helpless; they look to us naturally for acts of tenderness and love toward them; and if, instead, they should meet an over-share of sternness, of an unrelenting nature, the heart is soon wounded, the affections chilled, their trustingness crushed, and the foundation is laid for a spirit of enmity, dislike, and actual casting off of the tie which binds to home and all its endearments; and when that is once fairly done, the mischief is without remedy in all time thereafter. Let parents bear these things in mind. A fitful recognizance of their truthfulness is not sufficient; the impression should be of an abiding character, for none other will be sufficient to restrain the promptings of an impetuous nature or of a hasty temperament; thus is it that, in a moment, sometimes an act has been committed or a word uttered, laying the foundation for life-long remorses.

